# Management of *Mycobacterium avium* subsp. *paratuberculosis* in dairy farms*:* Selection and evaluation of different DNA extraction methods from bovine and buffaloes milk and colostrum for the establishment of a safe colostrum farm bank

**DOI:** 10.1002/mbo3.875

**Published:** 2019-08-17

**Authors:** Fabrizio Gamberale, Gabriele Pietrella, Marcello Sala, Paola Scaramella, Silvia Puccica, Valeria Antognetti, Norma Arrigoni, Matteo Ricchi, Antonella Cersini

**Affiliations:** ^1^ Istituto Zooprofilattico Sperimentale del Lazio e della Toscana M. Aleandri Rome Italy; ^2^ Istituto Zooprofilattico Sperimentale della Lombardia e dell’Emilia Romagna, National Reference Centre for paratuberculosis Podenzano Italy

**Keywords:** Colostrum, DNA extraction, evaluation, MAP, Real‐time PCR

## Abstract

The aim of this study was to develop and validate different innovative DNA extraction methods to detect *Mycobacterium avium* subsp. *paratuberculosis (MAP)* DNA from bovine and buffalo colostrum. Paratuberculosis is a chronic inflammatory infection of domestic and wild animals, especially ruminants, caused by MAP. The primary route of disease transmission is feces, but MAP can also be excreted in milk and colostrum. In 2015, the Italian Ministry of Health has issued a voluntary control plan of MAP in order to allow risk‐based certification of bovine and buffaloes farms. In addition to the annual diagnostic screening and to the clinical surveillance of animals the plan includes the adoption of biosecurity and management measures to progressively mitigate the incidence of MAP. To achieve this goal it is crucial to ensure the accuracy of the methods used to detect the presence of MAP in bovine and buffaloes milk and colostrum, in order to: (1) support a "safe colostrum farm‐bank" set‐up and thus prevent the main within‐farm MAP transmission route and (2) to allow the MAP‐free certification of milk products for export purposes. To achieve these goals, seven different DNA extraction protocols were identified from bibliography, out of which three methods were finally selected after the adoption of an evaluation procedure aimed at assessing the efficiency of extraction of DNA, the purity of DNA and the adaptability of the DNA amplification: NucleoSpin^®^ Food Kit (Macherey‐Nagel), NucleoSpin^®^ Food Kit (Macherey‐Nagel) combined with the magnetic beads, and QIAamp Cador Pathogen Mini kit (QIAGEN). In particular, the NucleoSpin^®^ Food Kit (Macherey‐Nagel) and the QIAamp Cador Pathogen Mini kit (QIAGEN) were tested on bovine and buffalo colostrum, showing a LOD between 4 × 10^4^ (2.6 × 10^6^ cfu/ml) and 4.08 (26.7 cfu/ml) IS900 target copies and a LOD between 5.3 × 10^5^ (4.1 × 10^6^ cfu/ml) and 53 (4.1 × 10^3^ cfu/ml) IS900 target copies, respectively.

## Graphical abstract

The aim of this study was to validate different DNA extraction methods for detection Mycobacterium avium subsp. paratuberculosis (MAP) DNA from bovine and buffalo colostrum to support a "safe colostrum farm bank" setup and thus prevent the main within farm MAP transmission route, as well as to allow the MAP free certification of milk products for export purposes. Two protocols were considered completely accurate: NucleoSpin Food Kit and QIAamp Cador Pathogen Mini kit.

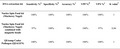

## 1 INTRODUCTION

*Mycobacterium avium* subsp. *paratuberculosis* (MAP) is the etiological agent of paratuberculosis (Johne's Disease), a contagious and chronic gastrointestinal disease affecting domestic and wild ruminants. The disease has a primarily oral‐fecal transmission route and the main source of infection is represented by feces and milk of the affected animals. In addition, there is the possibility of a bacterial streaming in other body districts, such as womb, testicular parenchyma, and breast representing another source of dissemination (Ayele, Svastova, Roubal, Bartos, & Pavlik, 2004; Sweeney, Whitlock, & Rosenberger, 1992; Whittington & Windsor, 2009). After the infection, the disease can remain in a subclinical phase for several years, and only few animals show typical symptoms of paratuberculosis (Klinkenberg & Koets, 2015). The MAP excretion is maximal during the clinical phase and the peak of disease spread through the milk and colostrum is observed during the last stages of the disease (Bakker, Willemsen, & Zijderveld, 2000; Streeter, Hoffsis, Bech‐Nielsen, Shulaw, & Rings, 1995; Sweeney, 2011; Windsor & Whittington, 2010; Zervens, Nielsen, & Jungersen, 2013). The colostrum represents a vital source for calves, containing maternal immunoglobulins (IgG). Calves are agammaglobulinemic at birth and acquire their immunity through absorption of IgG presents in colostrum. Furthermore, colostrum contains essential elements such as growth factors, maternal cells, and vitamins (Gauthier, Pouliot, & Maubois,^2006^; Godden, 2008; Houser, Donaldson, Kehoe, Heinrichs, & Jayarao,^2008^). However, the MAP bacteria were demonstrated to be present in the colostrum of subclinical and clinical cows by PCR between 24 ± 12 cfu/ml (2.4 ± 1.2 × 10^1^ cfu/ml) and 254 ± 63 cfu/ml (2.54 ± 6.3 × 10^2^ cfu/ml) (Stabel, Bradner, Robbe‐Austerman, & Beitz, 2014; Verhegghe, 2017) and the intake of colostrum contaminated by MAP represents one of the main routes of transmissions of infection for young calves. The MAP has been found in commercial cow's milk for human consumption and might represents a risk to public health due to its possible relationship with Crohn's disease (Ayele et al., 2004; Carvalho, Pietralonga, Schwarz, Faria, & Moreira, 2012; Donaghy et al., 2008; Ellingson, Anderson, & Koziczkowski, 2005; Grant, Ball, & Rowe, 1998, 2002; Paolicchi, Cirone, Morsella, & Gioffré, 2012; Shankar et al., 2009; Slana, Paolicchi, Janstova, Navratilova, & Pavlik, 2003). Moreover, many reports refer to the presence of MAP DNA in powdered infant formula (Hruska, Bartos, Kralik, & Pavlik, 2005, Donaghy, Johnston, & Rowe, 2010, Hruska, Slana, Kralik, & Pavlik,^2011^). The serological ELISA test on bovine and buffalo serum is usually used as a screening test, because it is a rapid and low‐cost assay, but its sensitivity is very low (15%) during the subclinical stage (Chui, King, & Sim, 2010). The cultural isolation is considered the gold standard in the detection of MAP, but it is time consuming especially in the case of samples with a low number of viable bacteria, for which long incubation times are needed to obtain bacterial growth. However, the sensitivity of this test in the animals affected by subclinical infection is reduced (23%–29%), while the specificity is up to 100%. (Nielsen & Toft, 2008). On the other hand, PCR represents a rapid test to detect MAP in feces, milk, and clinical samples (Fang et al., 2002; Grant et al., 1998; Millar et al., 1996; O'Mahony & Hill, 2002; Pillai & Jayarao, 2002). The most common molecular target for this assay is the IS900, an insertion element present in 15–18 copies in MAP genome (OIE, 2014; Ricchi et al., 2016). However, the detection of IS900‐like sequences in non‐MAP isolates represents a possible cause of false‐positive results (Green, 1993; Laurin, McKenna, Chaffer, & Keefe, 2015; Taddei et al., 2008; Tasara, Hoelzle, & Stephan, 2005). To overcome the problem, single‐copy F57 target has been used to confirm the IS900 assay from different matrix, including milk (Meadus, Gill, Duff, Badoni, & Saucier, 2008; Ricchi et al., 2009; Schonenbrucher, Abdulmawjood, Failing, & Bulte, 2008; Stephan, Schumacher, Tasara, & Grant, 2007; Tasara et al., 2005). Since the global objective is to progressively mitigate the risk of incidence of paratuberculosis infections in cattle and buffaloes and possibly reduce the exposure of humans to MAP through the food chain, it is crucial to ensure the accuracy of the methods used to detect its presence in bovine and buffaloes milk and colostrum. In this scenario, the availability of accurate methods and protocols aimed at establishing a safe colostrum farm‐bank, representing an important step to prevent one of the main whitin‐farm MAP transmission route. In Italy, the Ministry of Health set off in 2015 a voluntary control plan of MAP in order to allow a risk‐based certification of both bovine and buffaloes farms and products also for export purposes (SANCO, UE, European Commission, 2000; Veterinary certificate for import of milk and milk products into India, IN‐LO1; Health Certificate for milk, milk products intended to be exported from Italy to people's Republic of China, RPC‐LO1). In addition to the annual diagnostic screening and to the clinical surveillance of animals the plan includes the adoption of biosecurity and management measures to progressively mitigate the incidence of MAP. The aim of this study was to set up and validate different innovative DNA extraction methods to detect MAP DNA from bovine and buffalo milk and colostrum. In the first stage of the work, seven different DNA extraction methods have been tested on artificially contaminated pasteurized commercial milk, selecting the three best protocols according to the yield and the bibliographic research. Then, experiments on artificially contaminated and negative bovine and buffalo milk and colostrum samples have been conducted aimed at the performance evaluation of the selected extraction protocols through the interrater agreement assessment; finally, the protocols were tested on buffalo colostrum field samples and interrater agreement was again assessed.

## 2 MATERIALS AND METHODS

### 2.1 Materials

The protocol was validated using the *Mycobacterium avium* subsp. *paratuberculosis* (ATCC 19689) and the TOPO‐TA‐IS900 recombinant plasmid (TOPO TA Cloning^®^ Kit Dual Promoter pCR^®^II‐TOPO^®^ vector 45‐0640, Invitrogen, Carlsbad, CA).

A three‐step evaluation framework was applied.

### 2.2 Step 1 ‐ DNA extraction kits selection and LOD verification on commercial pasteurized Milk

#### 2.2.1 Artificial contamination of milk

Two liters of commercial pasteurized bovine milk were purchased from a local supermarket, then aliquotated in 50 ml test tubes and stored at −20°C until analysis.

The aliquots were thawed at room temperature and half of them were artificially contaminated with 1.15 × 10^5^ cfu/ml MAP strain (ATCC 19689), grown on Herrold's egg yolk medium for 3 months at 37°C.

The remaining aliquots were contaminated with 3.6 × 10^11^ TOPO‐TA‐IS900 recombinant plasmid, corresponding to 3.1 × 10^10^ cfu/ml.

The two groups of aliquots have been subjected to two different analyses for the DNA extraction kit selection and the LOD verification.

#### 2.2.2 DNA extraction kit

On the basis of the bibliographic research seven different DNA extraction protocols were selected, using commercial DNA extraction kits and modified commercial DNA extraction kits. In particular, the following protocols were selected: Chelex 100 (A), Chelex 100 sodium form (Fluka, Sigma‐Aldrich, 95577‐100G‐F, St. Louis, USA) combined with magnetic beads (provided by ADIAPURE^TM ^PARATB MILK kit, NEO4M1‐05, ADIAGENE, Ploufragan, France) and buffer L0 (provided by ADIAPURE^TM ^PARATB MILK kit, NEO4M1‐05, ADIAGENE, Ploufragan, France kit) (B), kit QIAMP DNA Blood Mini kit (QIAGEN, 51106, Hilden, Germany) (C), kit NucleoSpin^®^Food (Macherey‐Nagel, 740945.50, Dürer, Germany) (D), kit Nucleo Spin Food (Macherey‐Nagel, 740945.50, Dürer, Germany) combined with magnetic beads and buffer L0 (provided by ADIAPURE^TM ^PARATB MILK kit, NEO4M1‐05, ADIAGENE, Ploufragan, France kit) (E), Nucleo Spin Food (ADIAPURE^TM ^PARATB MILK kit, NEO4M1‐05, ADIAGENE, Ploufragan, France) combined with magnetic beads (provided by ADIAPURE^TM ^PARATB MILK kit, NEO4M1‐05, ADIAGENE, Ploufragan, France) (F), QIAMP Cador Pathogen Mini kit (QIAGEN, 54104, Hilden, Germany) (G) (Odumeru, Gao, Chen, Raymond, & Mutaria, 2001, Jay, Loessner, & Golden, 2005, Schlederer & Raberger, 2005, Slana, Liapi, Moravkova, Kralova, & Pavlik, 2009, Gazouli et al., 2010, Szteyn, Wiszniewska‐Laszczych, & Smolińska, 2014, Usman, Yu, Liu, Fan, & Wang, 2014, Volk et al., 2014, Di Pinto et al., 2007). All the methods were tested on pasteurized commercial bovine milk purchased from a local supermarket and contaminated with MAP strain (ATCC 19689). Then, the kits were selected on the basis of the DNA yield and purity evaluated through photometric reading and the Real time PCR results. (See Table 1).

In addition, further parameters for the kit selection were considered: the existence of validation and citation in literature about milk and colostrum extraction. Three DNA extraction protocols were selected, on the basis of the obtained results, discarding the methods that were widely cited in literature or methods for which poor performances had already been reported. The final selection of protocols included: (D) kit Nucleo spin Food; (F) kit Nucleo Spin Food combined with magnetic beads; (G) QIAamp Cador Pathogen Mini kit.

**Table 1:** Criteria for the DNA extraction kit selection

	Average yield	OD_260/280_	Real time PCR (average Ct)
A (1:10 diluted sample)	400 ng/μl	1.75	30.5
B (1:10 diluted sample)	400 ng/μl	1.00	28.6
C (undiluted sample)	10 ng/μl	0.9	26.4
D (undiluted sample)	21.5 ng/μl	1.75	21.8
E (undiluted sample)	13.5 ng/μl	1.00	23.7
F (undiluted sample)	18.15 ng/μl	1.67	20.8
G (undiluted sample)	21.5 ng/μl	1.75	21.5

#### 2.2.3 DNA extraction protocols

For the three selected protocols, 50 ml of commercial pasteurized milk was centrifugated at 2,500*g* for 20 min. The supernatant was discarded and the pellet was used for the DNA extraction.

- *Protocol (D): NucleoSpin^®^ Food kit (Macherey‐Nagel, 740,945.50, Dürer, Germany)*

The pellet was resuspended in 550 µl of Buffer CF. The sample was added to a 2 ml tube containing 10 µl of Proteinase K and incubated at 65°C for 30 min. The sample was centrifugated at 10,000g for 10 min, and then the DNA extraction was proceeded with kit instruction. Finally, the sample was eluted with 100 µl of elution buffer.

- *Protocol (F): NucleoSpin^®^ Food kit (Macherey‐Nagel, 740,945.50, Dürer, Germany) combined with magnetic beads (ADIAGENE, Ploufragan, France)*

The pellet was resuspended in 50 µl of magnetic beads (Adiapure) and incubated in continuous stirring at room temperature for 30 min. The pellet was recovery putting the sample on magnetic support for 20 min. After, the liquid phase was discarded and the sample was resuspended with 550 µl of CF Buffer and 10 µl of proteinase K in a 2 ml tube. Then, the solution was incubated at 65°C for 30 min and the DNA extraction was proceeded with kit instruction. Finally, the sample was eluted with 100 µl of elution buffer.

- *Protocol (G): QIAamp Cador Pathogen Mini Kit (QIAGEN, 51,106, Hilden, Germany)*

The pellet was added to a 2 ml tube containing 100 µl of VXL buffer and 10 µl of proteinase K and the sample was incubated at 25°C for 15 min. The DNA extraction was proceeded with kit instruction. Finally, the sample was eluted with 100 µl of elution buffer.

#### 2.2.4 Real Time IS900

The master mix was composed by: 10 μl of Buffer Master Mix 2X (TaqMan^® ^Universal PCR Master Mix, Applied Biosystems, Life Technologies, 4,318,157, Warrington, UK), 0.2µl [30 µM] forward and reverse primers, 0.3 µl [10 µM] TaqMan probe (see Table 2), 3.1µl DEPC water, 5 μl of DNA template, 1 µl of 0.5X Exogenous Internal Positive Control‐IPC (Universal Exogenous qPCR Positive Control (Yakima Yellow‐TAMRA probe), RT‐IPC‐TO2, Eurogentec, Seraing, Belgium), 0.2 of 0.5X IPC buffer (Universal Exogenous qPCR Positive Control (Yakima Yellow‐TAMRA‐probe), RT‐IPC‐TO2, Eurogentec, Seraing, Belgium), in a final volume of 20 µl. The IPC control was added in order to detect possible false‐negative results due to PCR inhibition. Real time PCR was conducted using ABIPRISM 7900 HT Sequence Detection System (Applied Biosystems) and using the following thermal profile: 95°C for 10 min, 50 cycles of 95°C for 15 s, and 60°C for 1 min. The amplification protocol was obtained by the National Reference Centre for paratuberculosis.

**Table 2:** Primers and probe sequences used in Real Time PCR IS900 to detect MAP

Oligonucleotide	5′‐3′ sequence
IS900 Forward Primer	CCGGTAAGGCCGACCATTA
IS900 Reverse Primer	ACCCGCTGCGAGAGCA
IS900 Probe	6 FAM‐CATGGTTATTAACGACGACGCGCAGC‐TAMRA

#### 2.2.5 Limit of detection (LOD) for applicability of selected molecular protocols.

For each considered matrix two standard curves were analyzed. The first was drawn up using the ATCC 19689 MAP strain, while the second using the recombinant TOPO‐TA plasmid containing IS900 target (TOPO‐TA‐IS900) (TOPO TA Cloning^®^ Kit Dual Promoter pCR^®^II‐TOPO^®^ vector 45‐0640, Invitrogen, Carlsbad, CA). The standard curves were obtained from the linear regression line through the data points on a plot of Ct versus the logarithm of the internal standard concentration. Both the obtained standard curves showed values between 5.3 × 10^10^ target copies (equivalent to 4.1 × 10^11^ cfu/ml), and 5.3 IS900 target copies (equivalent to 4.1 × 10^1^ cfu/ml), with corresponding Ct values between 3.6 × 10^11^ target copies (equivalent 3.1 × 10^10^ cfu/ml) and 3.6 IS900 target copies (equivalent to 2.4 × 10^1^ cfu/ml). In addition, LOD were verified for all three selected DNA extraction protocols using three different operators.

### 2.3 Step 2 ‐ Performance evaluation of the selected protocols on bulk tank milk and individual colostrum

Bovine and buffalo bulk tank milk and individual colostrum were taken from two farms periodically tested for MAP and always negative (ELISA on individual blood samples, bacteriological examination and Real Time PCR on feces) since 2014 and thus considered free from infection.

In addition, each milk or colostrum sample collected on farm was aliquotated in 50‐ml test tubes on arrival at the Laboratory, then subjected to both Real‐Time PCR and bacteriological examination in order to assess the absence of MAP. Finally the samples were stored at −20°C until analysis.

Milk and colostrum samples were thawed at room temperature and artificially contaminated at increasing concentrations with TOPO‐TA‐IS900 recombinant plasmid.

#### 2.3.1 Panels constitution.

Two different panels of milk and colostrum samples were constituted as follows:

- 10 negative (non contaminated) samples.
- 10 positive samples artificially contaminated with 5.3 TOPO TA‐IS900 target copies (corresponding to 4.1 × 10^1^ cfu/ml).
- 10 positive samples artificially contaminated with 5.3 × 10^5 ^TOPO‐TA‐IS900 target copies (corresponding to 4.1 × 10^6^ cfu/ml).
- 10 positive samples artificially contaminated with 5.3 × 10^10 ^TOPO‐TA‐IS900 target copies (corresponding to 4.1 × 10^11^ cfu/ml).

The milk and colostrum panels were tested in triplicate with each of the three different selected extraction protocols and using three different operators, for a total of 120 samples/protocol.

Each operator performed the following workflow: (1) DNA extraction of the 30 contaminated colostrum sample; (2) DNA extraction of the 10 negative colostrum samples; (3) for each extracted DNA the purity was verified through the photometric quantification; (4) each DNA extraction was processed in double as template for q Real‐Time PCR IS900.

#### 2.3.2 Evaluation schedule

Through the evaluation process the following statistic parameters were calculated: sensitivity (Se) by determining the LOD, specificity (Sp), accuracy, positive predictive value (VPP), negative predictive value (VPN), and the statistic K value (Cohen concordance), based on the OIE manual (OIE, 2008, 2013).The sensitivity was calculated according to the formula:

Se=TP/TP+FP.

The specificity was calculated according to the formula:

Sp=TN/TN+FP.

The accuracy was calculated according to the formula:

Accuracy=TP+TN/TP+TN+FN+FN.

The positive predictive value was calculated according to the formula:

VPP=TP/TP+FP.

The negative predictive value was calculated according to the formula:

VPN=TN/FN+TN.

where “TP” represents true positive, “FP” represents false positive, “TN” represents true negative and the “FN” represents false negative according to test results of the standard assay of comparison.

The statistic *K* value was calculated considering that *K* > 0.75 indicates an optimum concordance level between multiple operators not attributable to the impact of the case. For the evaluation was used the following formula:

$$K=P_{0}-P_{e}/1-P_{e}.$$

where** “** *P*_0_” represents the sum of observed concordance rate and “*P*_e_” represents the sum of the products of the marginal level of percentages.

The values of *K* test were established on the basis of procedures reported on the OIE manual, in which the optimal *K* value is between 0.81 and 1 (OIE, 2008; Cap.1, 27–43).

### 2.4 Step 3 ‐ “On‐field evaluation” of the selected protocols on individual buffalo colostrum

Two liters of buffalo individual colostrum were collected from two naturally infected but asymptomatic animals, 1 L each. The animals were classified infected as they resulted positive to ELISA test on blood and to bacteriological examination and to Real‐Time PCR on feces.

Two liters of buffalo individual colostrum were collected from two clinically healthy noninfected animals (ELISA negative and negative to bacteriological examination and to Real‐Time PCR on feces) from a farm MAP free since 2014.

All samples were aliquotated in 50‐ml test tubes and stored at −20°C until analysis.

Milk and colostrum samples were thawed at room temperature.

Before each experiment, a bacteriological analysis was performed on each sample in order to evaluate possible interferences with the results due to the presence of other pathogenic or commensal microorganisms (*Enterobacteriaceae*, *Streptococcaceae*, *Listeria Monocytogenes*, *Pseaudomonas* spp., sulfite‐reducing clostridia).

A panel of buffalo colostrum samples was constituted as follows:

- 10 negative samples from clinically healthy and tested negative animals (blood and feces)
- 10 positive samples from naturally infected and tested positive animals (blood and feces)

The panel was tested in triplicate with each of the three different selected extraction protocols and using three different operators, for a total of 60 samples/operator.

## 3 RESULTS

### 3.1 **S**tep 1 ‐ Selection of DNA extraction protocols on pasteurized milk

The photometrical reading reported a yield of 21.5 ng/µl and a DNA purity of 1.75 for the Cador Pathogen kit, a yield of 21.5 ng/µl and a DNA purity of 1.75 for Macherey‐Nagel kit and a yield of 18.15 ng/µl and a DNA purity of 1.67 for Macherey‐Nagel kit combined with magnetic beads.

All other methods were discarded because of a yield less than 15 ng/µl and a low DNA purity level (~0.8) (ratio od_260/280_). In particular, the evaluated LOD for the three selected extraction protocols were between 1.15 × 10^5^ (corresponding to 1.9 × 10^6^ cfu/ml) and 2 IS900 target copy (corresponding to 20 cfu/ml) and 3.6 × 10^6 ^(corresponding to 2.7 × 10^7^ cfu/ml) and 3.7 IS900 target copy (26 cfu/ml), respectively.

### 3.2 Step 2 – Performance evaluation of the selected protocols on artificially contaminated bovine and buffalo bulk tank milk and individual colostrum

The best yield for the bovine bulk tank milk was of 15 ng/µl for Macherey‐Nagel kit.

The QIAamp Cador Pathogen Kit showed a yield level slightly below (13 ng/µl).

Similarly, the best LOD was obtained with Macherey‐Nagel kit, with a range from 4.11 × 10^8 ^(2.3 × 10^9^ cfu/ml) to 3.9 (26 cfu/ml) IS900 target copies.

For the buffalo bulk tank milk the best photometric yield was obtained with Macherey‐Nagel kit, reaching a value equal to 46.38 ng/µl; in this case the LOD was between 3.7 × 10^8 ^(2.6 × 10^9^ cfu/ml) and 3.82 (25 cfu/ml) IS900 target copies.

In both bovine and buffalo samples part of magnetic beads remained trapped in the milk fat component, reducing the total extraction yield. The presence/absence of somatic cells in the samples was also analyzed, observing that their presence can hinder the beads recovery and consequently the extraction yield. However, the Macherey‐Nagel kit presents a pretreatment buffer that effectively reduces the cellular and fat component.

Analogous results were obtained for bovine and buffaloes colostrum, related to the extraction yield both for the Macherey‐Nagel kit and for the Macherey‐Nagel kit combined with magnetic beads (22.45 ng/µl). In addition, the LOD obtained from bovine colostrum resulted equivalent for both DNA extraction kits, with a range values from 4 × 10^4 ^(2.6 × 10^6^ cfu/ml) and 4.08 (26.7 cfu/ml) IS900 target copies.

The QIAamp Cador Pathogen kit for the bovine colostrum showed a lower yield extraction equal to a 10 ng/µl and a LOD with a range value from 4 × 10^3 ^and 10 IS900 target copy.

Regarding the extraction from buffalo colostrum, the Macherey‐Nagel kit showed an evident great yield value (16.4 ng/µl) compared with Macherey‐Nagel kit combined with the magnetic beads (10 ng/µl).

For the QIAamp Cador Pathogen kit were reported only the results on the buffalo and bovine colostrum.

This result is due to the high presence of fat that traps a large portion of magnetic beads.

To recover a significant quantity of magnetic beads, the centrifugation phase has been eliminated, as the beads remain concentrated at the bottom of the tube, together with the colostrum fat particles, making it impossible to obtain a good magnetic separation.

The best LOD resulted for Macherey‐Nagel kit, between 4.28 × 10^5 ^(2.8 × 10^6^ cfu/ml) and 3.91 (26.6 cfu/ml) IS900 target copy. QIAamp Cador Pathogen showed a less yield extraction, especially for buffalo colostrum. In fact, contaminating 90 samples with TOPO‐TA IS900, the LOD resulted between 5.3 × 10^5 ^(4.1 × 10^6^ cfu/ml) and 53 (4.1 × 10^3^ cfu/ml) IS900 target copies for all the experiments with three operators. (see Tables 3 and 4).

**Table 3:** Sperimental data obtained from bovine and buffalo colostrum on contaminated field samples

Kit	Matrix	Average yield	LOD IS900 target copy/μl	LOD UFC/ml
Nucleo Spin Food kit (Macherey Nagel)	Bovine milk	15 ng/μl	4.11 × 10^8^−3.9	2.3 × 10^9^−26
Nucleo Spin Food kit (Macherey Nagel)	buffalo milk	46.38 ng/μl	3.7 × 10^8^−3.82	2.6 × 10^9^ −3.82
Nucleo Spin Food kit (Macherey Nagel) combined with magnetic beads	Bovine milk	10 ng/μl	1.7 × 10^7^−7.7	1.75 × 10^9^−7.7
Nucleo Spin Food kit (Macherey Nagel) combined with magnetic beads	buffalo milk	47.6 ng/μl	3.86 × 10^6^−9.7	2.54 × 10^9^−5

**Table 4:** Sperimental data obtained from bovine and buffalo milk on contaminated field samples

Kit	Matrix	Average yield	LOD IS900 target copy/μl	LOD UFC/ml
Nucleo Spin Food kit (Macherey Nagel)	Bovine colostrum	22.45 ng/μl	4 × 10^4^−4.08	2.6 × 10^6^−26.7
Nucleo Spin Food kit (Macherey Nagel)	buffalo colostrum	16.4 ng/μl	4.28 × 10^5^−3.91	2.8 × 10^6^−26.6
Nucleo Spin Food kit (Macherey Nagel) combined with magnetic beads	Bovine colostrum	22.45 ng/μl	4.11 × 10^5^−4.13	1.33 × 10^6^−13.8
Nucleo Spin Food kit (Macherey Nagel) combined with magnetic beads	buffalo colostrum	10 ng/μl	4.11 × 10^5^−4.13	1.33 × 10^5^−13.8
Qiamp Cador Pathogen (QIAGEN)	Bovine colostrum	10 ng/μl	4 × 10^3^−10	4.1 × 10^6^−1.2 × 10^4^
Qiamp Cador Pathogen (QIAGEN)	buffalo colostrum	10 ng/μl	5.3 × 10^5^−53	4.1 × 10^6^−4.1 × 10^3^

The statistical results of selected DNA extraction methods from bovine and buffalo colostrum were reported (see Table n.5).

The Macherey‐Nagel kit without magnetic beads and QIAamp Cador Pathogen kit showed perfect statistic values, evidencing 100% sensitivity and specificity with a *K* value equal to 1, underlining an optimal concordance between operators.

The Macherey‐Nagel kit with magnetic beads gave a 100% specificity and only 97.7% sensitivity due to the misclassification by two operators of two positive samples as negative at a 5.3 × 10^5 ^TOPO‐TA‐IS900 target copies (4.1 × 10^6^ cfu/ml) concentration in the buffaloes colostrum panel.

**Table 5:** Comparision table of statistical parameters for DNA extraction selected kit on bulk tank bovine/buffalo milk and individual bovine/buffalo colostrum

DNA extraction kit	Sensitivity %^a^	Specificity %^b^	Accuracy %^c^	VPP %^d^	VPN %^e^	K value^f^
Nucleo Spin Food kit (Macherey Nagel)	100%	100%	100%	100%	100%	1
Nucleo Spin Food kit (Macherey Nagel) combined with magnetic beads	97%	100%	98%	100%	97%	0,96
QIAamp Cador Pathogen (QIAGEN)	100%	100%	100%	100%	100%	1

a Measures the proportion of actual positives that are correctly identified as such.

b Measures the proportion of actual negatives that are correctly identified as such.

c Difference between sample average value and true or reference values.

d The probability that a positive sample is actually positive.

e The probability that a negative sample is actually negative.

f Statistical coefficient representing the degree of accuracy and reliability in a statistical classification.

### 3.3 Step 3 ‐ Results for individual buffalo colostrum (field samples)

For the on field evaluation of the selected protocols on individual buffalo colostrum, were considered 10 positive samples with the same level of MAP contamination (1.8 × 10^5^) and 10 negative samples for each operator. In the Table 6 was reported the panel constitution and the average Ct detected on the individual buffalo colostrum using three operators (see Table 6).

A 100% sensitivity and specificity resulted for the three selected protocols used. All positive and negative samples of the panel were correctly classified with a complete accordance (100%) between operators and between the selected protocols.

**Table 6:** On field evaluation of teh selected protocols on individual buffalo colostrum for each three operators

		Average Ct
Nucleo spin tissue (Macherey Nagel)	10 positive samples	22.68
	10 negative samples	0
Nucleo spin tissue (Macherey Nagel) combined with Magnetic beads	10 positive samples	23.24
	10 negative samples	0
Qiamp s kit (QIAGEN)	10 positive samples	23.21
	10 negative samples	0

## 4 DISCUSSION

The bibliographic review conducted on commercial and home‐made kits routinely used for the DNA extraction from complex matrices, rich in fats and proteins, allowed a preliminary selection of kits and protocols with the best theoretical potential for a further development and for their application to MAP DNA extraction from milk and colostrum.

Specifically, the DNA extraction protocols subjected to evaluation were those routinely employed to detect GMO in different foods, such as chocolate products, cocoa, nougat products, breakfast cereals, muesli, nut/chocolate spread, jam and fruit concentrates, pollen, lecithin, spices, bread, raw processed products and cosmetics (plant and animal ingredients).

The evaluation process (step 1) allowed to verify the actual suitability of three protocols for MAP DNA extraction from the matrix “milk,” confirming their potential validity of application on bovine and buffalo milk and colostrum taken in the field.

The results obtained from the evaluation process on artificially contaminated bulk tank milk and individual colostrum revealed (step 2), for two protocols out of three, 100% sensitivity and specificity for MAP DNA extraction at increasing concentration of TOPO‐TA‐IS900 recombinant plasmid, along with a perfect repeatability between operators.

Two protocols were therefore considered completely accurate: Nucleo Spin Tissue kit (Macherey‐Nagel), with LOD between 4 × 10^4 ^(2.6 × 10^6^ cfu/ml) and 4.08 (26.7 cfu/ml) IS900 target copies (bovine colostrum); QIAamp Pathogen Cador kit with LOD between 5.3 × 10^5^ (4.1 × 10^6^ cfu/ml) and 53 (4.1 × 10^3^ cfu/ml IS900 target copies (buffalo colostrum).

Both LOD detected with the two selected protocols were within the range of bacterial count observed in dairy cattle with subclinical symptoms (Stabel et al., 2014), specifically between 1.24 × 10^4 ^and 1.4 cfu/ml.

However, the accuracy values of the DNA extraction methods resulted partly influenced by the presence of the fat component in milk. In particular, this factor could have hampered the recovery of magnetic beads which may have determined lower Sensitivity, VPN and K values for the third selected protocol, the Macherey‐Nagel kit with magnetic beads.

All the positive (*N* = 90) and negative (*N* = 30) samples within the bovine and buffaloes bulk tank milk panels were correctly classified by the three operators using this latter protocol (Macherey‐Nagel protocol with magnetic beads). Nevertheless only two false negative results, both relative to the 5.3 × 10^5 ^TOPO‐TA‐IS900 target copies contamination dose (corresponding to 4.1 × 0^6^ cfu/ml, out of 90 total positive samples were provided by two operators within the buffaloes colostrum panel.

As a consequence, this protocol showed an overall 100% specificity and a lower sensitivity (97.7%), with an interoperators agreement classified as “good” (K statistic value = 0.966).

Furthermore, it cannot be excluded that the two occurred misclassifications may have been determined by two gross errors by the two operators.

However, the presence of fat particles in the samples was considered critical because of the possible technical interferences on DNA extraction. In order to verify the stability of the protocols’ performance in field conditions, a challenge was performed involving the Real‐Time PCR MAP diagnosis on colostrum samples from asymptomatic tested positive and clinically healthy tested negative buffaloes (step 3), in a worst scenario due to the higher concentration of fats in this matrices compared to bovine milk or colostrum.

From this experiment, all the samples in the panel from asymptomatic infected animals were classified as positive, with a complete accordance (100%) between operators, thus suggesting the “on‐field” validity of all three protocols.

In light of the results obtained and based on the above reported considerations, it's authors’ opinion that the Macherey‐Nagel protocol with magnetic beads without the centrifugation step should also be considered as accurate as the other two protocols.

Despite the results obtained demonstrated the validity of the protocols for a routine MAP diagnosis in bovine and buffaloes milk and colostrum, accuracy assessment needs additional improvement, specifically on field samples from infected animals during the subclinical phase, when lower concentrations of MAP DNA in milk and colostrum are likely to occur.

However, it is noteworthy that a 100% accuracy resulted in this study for the milk and colostrum samples artificially contaminated at the lowest concentration of TOPO‐TA‐IS900 recombinant plasmid (5.3 TOPO TA‐IS900 target copies corresponding to 4.1 × 10^1^ cfu/ml), near to the minimum MAP bacterial count observed in the milk of animals during the subclinical or clinical phase of paratuberculosis (Stabel et al., 2014).

The validity of the three selected protocols prefigures an innovative approach to the diagnosis of MAP infection in bovine and buffaloes milk/colostrum.

At technical level, the protocols could offer the advantage of reducing the inhibitor effect of the fat component on MAP DNA extraction, thus increasing the sensitivity and the VPN of the analysis for fat reach matrices.

Colostrum represents a risk factor for the transmission of several diseases and many dairy farms rely on the creation of a safe colostrum bank in order to hamper the possible spread of diseases at weaning. To prevent one of the main MAP transmission route for young calves it is crucial to ensure an accurate analysis of individual colostrum, in order to feed the animals with a safe and certificated MAP free source of nutrition.

Based on the results obtained from this study, a protocol for a safe colostrum farm bank could be suggested, consisting in the collection of colostrum sample in plastic bottles from every single bovine/buffalo resulted negative to the serological screening ELISA test. Each bottle must be identified with a univocal identification number and should be stored at −20°C on farm, waiting for the laboratory analysis. Before freezing, 100/200 ml of colostrum from every bottle must be collected and immediately sent to the laboratory for analysis in freezing condition. The samples are kept at 4°C before the test. The sample resulted positive for MAP DNA PCR must be discarded, and the negative samples could be stored in the colostrum farm bank.

Another practical advantage of the validated protocols is represented by the possibility to analyze pool of samples, reducing both the times needed for the diagnosis and the costs for the breeders

The availability of accurate PCR protocols for the diagnosis of MAP on milk and colostrum can also contribute to extend the diagnostic effectiveness both at individual and at farm level, in combination with blood serology and bacteriological examination of feces.

Used as a complementary test, MAP DNA PCR on milk and colostrum can provide a useful support for paratuberculosis control programs (DCP) on farms, allowing an effective separation of lactating animals based on their health status and a targeted management of clinical and eventually subclinical shedders.

The systematic application of combined diagnostic protocols (serology, bacteriology on feces and Real‐Time PCR on feces, milk, and colostrum) along with the adoption of efficient biosecurity measures is the most effective strategy to progressively mitigate the incidence of MAP towards the risk‐based certification of both bovine and buffaloes farms, helping to reduce the potential risk of exposure of humans to MAP through the food chain.

Recently, agreements have been signed between Italy and some third countries, for example China and India, to ensure the export of dairy products certified free from several diseases, such as tuberculosis, brucellosis, anthrax, listeriosis, and paratuberculosis.

In this context, the use of the validated MAP DNA extraction protocols opens up realistic prospects for the specific certification of PDO dairy products, such as milk itself, Parmesan cheese, Pecorino Romano cheese, and buffalo's Mozzarella, also for export purposes.

## CONFLICT OF INTERESTS

None declared.

## AUTHOR CONTRIBUTIONS

Fabrizio Gamberale, Gabriele Pietrella, and Antonella Cersini were involved in data curation and visualization.

Fabrizio Gamberale was involved in conceptualization and funding acquisition.

Marcello Sala was involved in study design and formal analysis.

Antonella Cersini, Gabriele Pietrella, Valeria Antognetti, Silvia Puccica, and Paola Scaramella were involved in investigation.

Antonella Cersini was involved in methodology.

Fabrizio Gamberale and Antonella Cersini were involved in project administration and resources,

Norma Arrigoni and Matteo Ricchi were involved in supervision. Antonella Cersini, Gabriele Pietrella, Valeria Antognetti, and Silvia Puccica were involved in validation

Fabrizio Gamberale, Antonella Cersini, Gabriele Pietrella were involved in writing—original draft preparation.

Fabrizio Gamberale, Antonella Cersini, Gabriele Pietrella, Marcello Sala were involved in writing—review and editing.

## ETHICS STATEMENT

None required.

## Data Availability

All data generated or analyzed during this study are included in this published article and its supplementary information files.
